# Tremor Following Guillain Barré Syndrome

**DOI:** 10.5334/tohm.906

**Published:** 2024-10-25

**Authors:** José Fidel Baizabal-Carvallo, Carlos Manuel Cortés, Marlene Alonso-Juarez, Robert Fekete

**Affiliations:** 1 Department of Sciences and Engineering, University of Guanajuato, León, México; 2 Department of Internal Medicine, University of Guanajuato, León, México; 3 Instituto Politécnico Nacional, Mexico; 4 New York Medical College, Valhalla, New York, USA

**Keywords:** tremor, neuropathic, Guillain-Barré, syndrome, polyneuropathy, axonal

## Abstract

**Background:**

Neuropathic tremor occurs with damage to the peripheral nervous system. Guillain-Barré syndrome (GBS) causes acute paralysis following nerve inflammation sometimes resulting in long-term disability. It is unclear how frequent and severe tremor is following GBS.

**Objectives:**

We aimed to assess the frequency and features of tremor following GBS.

**Methods:**

We enrolled 18 patients with GBS treated in a secondary care center within a 4-year period. Evaluations were done with the Fahn-Tolosa-Marin tremor rating scale (FTM-TRS). We compared these features with a cohort of consecutive patients with untreated essential tremor (ET).

**Results:**

There were 13 males and 5 females with a mean age at evaluation (S.D.) of 41.5 ± 14.0 years and at GBS onset of 40.2 ± 13.7. No patient had history of tremor before GBS. Upper limb tremor was identified in 16 (89%) cases, 35.5% of patients had FTM-TRS score ≥10 points. Tremor was mostly kinetic, jerky with low amplitude with a total score of 10.94 ± 11.84 in the FTM-TRS. Compared with patients with ET, those with GBS-tremor were younger and had lower scores in all subscales of the FTM-TRS (*P* value < 0.05 for all comparisons). In a multivariate linear regression analysis “days of hospitalization” had a positive association with the total FTM-TRS score (*P* = 0.001).

**Conclusions:**

Tremor was common following GBS. This tremor is mild compared with patients with ET, but adds functional impact.

## Introduction

Tremor is one of the most common movement disorders. There are several types of tremors, including essential, parkinsonian, dystonic, Holmes, functional, etc. Among the etiologies causing tremor, damage to the peripheral nervous system is well-recognized. Patients with neuropathic tremor may present with postural, rest or kinetic tremor of the upper extremities with a 3–6 Hz frequency, diverse amplitude and not varying with weight loading [1,2]. Neuropathic tremor was initially recognized in patients with familial autosomal dominant Charcot-Marie-Tooth (CMT) neuropathy, the so-called Roussy-Lévy syndrome [3]. Neuropathic tremor was then identified in a proportion of patients with IgM paraproteinemic neuropathy, chronic inflammatory demyelinating polyradiculoneuropathy (CIDP), multifocal motor neuropathy, ischemic neuropathy, axonal neuropathy, etc. [4,5,6]. Although most patients with neuropathic tremor seem to have a related demyelination process, cases of axonal or mixed neuropathy have also been recognized [4,5,6]. However, there are relatively few reports on the frequency and severity of tremor in patients who have suffered an episode of acute polyneuropathy, i.e. Guillain-Barré syndrome.

Guillain-Barré syndrome (GBS) is one of the most common etiologies of acute flaccid paralysis, caused by an inflammatory response affecting the peripheral nervous system [7]. Although relatively rare, this disorder is consistently seen in general neurology or general hospitals with an estimated prevalence of 1–2 cases per 100,000 person/year [8]. Most patients start with weakness affecting the legs that progress in an ascending fashion eventually affecting the arms and cranial muscles. However, the clinical presentation may vary resulting in several clinical syndromes [9]. The diagnosis is primarily based on clinical history and neurological examination; whereas CSF findings and electrophysiology can support the diagnosis [7]. The latter is useful to determine the subtypes of GBS, including: acute inflammatory demyelinating polyradiculoneuropathy (AIDP), acute motor axonal neuropathy (AMAN) and acute motor sensory axonal neuropathy (AMSAN) [10].

## Materials and Methods

We studied consecutive patients who suffered axonal GBS in a secondary care center. We made a retrospective review of adult patients diagnosed with GBS assessed from January 2020 to December 2023. The Brighton criteria was used to determine the diagnostic certainty of GBS, level 1 (highest) to level 4 (lowest) [11]. These patients were selected from the hospital database and contacted by telephone for tremor and general neurological examination during the convalescent period. Patients still alive and willing to participate in the study were enrolled. The objective of the evaluation was omitted when patients were invited to the study.

Clinical and demographic variables were registered in the retrospective evaluation of the clinical features and course of GBS. Including age at onset, sex, presence, type and timing of a previous infection. The anatomical distribution of weakness was assessed, including cranial and respiratory involvement and the use of mechanical ventilation. We estimated the maximal weakness in upper and lower limbs by the Medical Research Council (MRC) scale [12]. We evaluated the type of neurological impairment, motor vs. motor/sensitive, supported by neurophysiology studies carried out during the acute phase of GBS. The maximal motor impairment was assessed by the Hughes functional grading scale as it follows, 0: healthy; 1: minor symptoms, able to run; 2: able to walk 10 meters or more without support, unable to run; 3, able to walk 10 meters with help; 4, bedridden or wheelchair bound; 5, requires assisted mechanical ventilation; 6, dead [13]. The modified Rankin Scale (mRS) was calculated at the patient admission and discharge. The type of treatment, either plasma exchange (PE) 5 sessions in alternate days or IV immunoglobulin (IVIg) 0.4 gr/Kg per day for 5 consecutive days were recorded. The residual weakness and functional impact were evaluated with the MRC scale and mRS, respectively, at the time of tremor assessment. The frequency of muscle atrophy secondary to denervation was also assessed. We evaluated the pharmacological therapy in all patients at the time of tremor assessment, in order to weight the potential contribution of tremorigenic medications (valproate, selective serotonin reuptake inhibitors (SSRIs), serotonin and norepinephrine reuptake inhibitors (SNRIs), tricyclic antidepressants) and medication with anti-tremorigenic potential (i.e., beta-blockers, topiramate, primidone, and gabapentin). In case any of these medications were taking at the time of tremor assessment, they were stopped, if possible, for at least 72 hours before tremor evaluation.

The clinical evaluation of tremor was carried out by means of the Fahn-Tolosa-Marin tremor rating scale (FTM-TRS) a widely used and validated scale [14]. The scale is composed by three parts with scores ranging from 0 (no tremor) to 4 (severe tremor). Part A evaluates tremor severity by clinical examinations in different body parts (maximum score: 80). Part B includes writing, drawing Archimedes spirals and pouring water (maximum score: 32) and part C includes items on tremor disability in daily activities (maximum score: 28), ranging from 0 (normal) to 4 (severely abnormal or severe impairment) [15]. Additionally, we assessed patients with untreated essential tremor (ET). These patients were consecutively evaluated in the same hospital and were not selected by their severity level. The diagnosis of essential tremor was based on the presence of bilateral, symmetric, upper limb kinetic tremor with a frequency between 4 and 12 Hz, not explained by other disorders [16]. We contrasted the demographic and clinical features of patients with ET and those with GBS tremor, in order to have a comparison reference to weight the degree of severity and functional impairment of GBS related tremor. All patients were evaluated by a movement disorders specialist (BC-JF). The local committee of ethics and research approved the study (no. PR01 AA) and patients or a close family member provided written informed consent to participate, the latter instance was in case the patient was unable to sign due to residual weakness.

## Statistics

We summarized data in means and standard deviations and percentages. The X2 and Fisher’s exact tests were used to compare nominal or ordinal data. The t-test was used to compare means between patients with previous GBS and patients with ET. A multivariate linear regression analysis was carried out to assess if independent variables predicted the final FTM-TRS score. Statistical evaluations were performed using SPSS version 22, a P value < 0.05 was considered significant.

## Results

**Figure 1:**
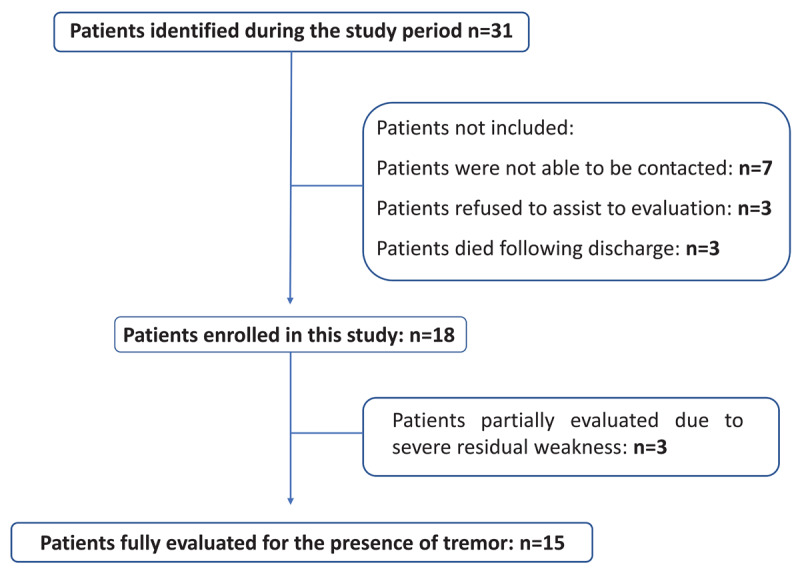
Summary of patients identified and enrolled in this study.

We identified 31 patients with a diagnosis of GBS within the study period. Thirteen patients were excluded due to multiple reasons (Figure 1). A total of 18 patients were enrolled and clinically evaluated for tremor and its functional impact. Three patients were partially evaluated for tremor owing to residual muscle weakness. However, all patients were evaluated for the presence of tongue, facial, voice and limb rest tremor.

The mean age at onset of GBS was 40.2 ± 13.7 years, and patients were assessed for tremor with a mean delay of 1.3 years following GBS onset. According to the Brighton criteria, there were 2 patients with level 1 (11.1%), 14 patients with level 2 (77.1%) and 2 patients with level 3 (11.1%). The majority of patients were male (72.2%). Thirteen patients had pure motor type of GBS and five patients had motor-sensory abnormalities. Seven patients underwent neurophysiology testing during the acute phase of GBS showing axonal type of GBS in all cases. Most patients had severe muscle weakness in the upper and lower extremities (MRC ≤ 2) at their peak severity; a mean maximal Hughes scale of 4.5; however, only 3 (16.7%) required mechanical ventilation during the acute phase of the disease. The clinical features related to the GBS are summarized in Table 1. Most patients were treated with IVIg (72.2%) and PE (22.2%), one patient did not receive pharmacological therapy for unclear reasons.

**Table 1:** Summary of clinical features of patients with Guillain-Barré syndrome.


	n = 18 (%)

Age at onset of GBS (years)	40.2 ± 13.7

Age at evaluation of tremor (years)	41.5 ± 14.0

Sex (males)	13 (72.2)

**Previous infection**	

Gastrointestinal	10 (55.6)

Respiratory	5 (27.8)

Time since infection to GBS onset (days)	10.2 ± 4.8

**Features of GBS**	

Craneal involvement	14 (77.8)

Respiratory impairment	4 (22.2)

Mechanical ventilation	3 (16.7)

Weakness peak severity in upper limbs (MRC)	

1	12 (66.7)

2	2 (11.1)

3	2 (11.1)

4	2 (11.1)

Weakness peak severity in lower limbs (MRC)	

1	12 (66.7)

2	3 (16.7)

3	2 (11.1)

5	1 (5.6)

Isolated motor impairment	13 (72.2)

Motor/sensitive impairment	5 (27.8)

Hughes scale (worst)	4.5 ± 1.1

**Management**	

Plasmapheresis	4 (22.2)

Intravenous immunoglobulin	13 (72.2)

No treatment	1 (5.6)

**Residual neurological impairment**	

mRS at discharge	3.33 ± 1.13

mRS at tremor evaluation	1.78 ± 1.0

Residual muscle weakness	

Cranial	0

Upper limbs	6 (33.3)

Lower limbs	5 (27.7)

Residual denervation, distal muscle hypotrophy	6 (33.3)


MRC: Medical Research Council Scale score; mRS: modified Rankin Scale.

At the time of tremor evaluation, patients had a mean mRS of 1.78 ± 1.0 points; 33.3% had residual weakness in the upper extremities and 33.3% had distal, mild upper or lower limb muscle atrophy secondary to denervation. Importantly, no patient or any of their family members endorsed tremor before the onset of GBS.

**Figure 2:**
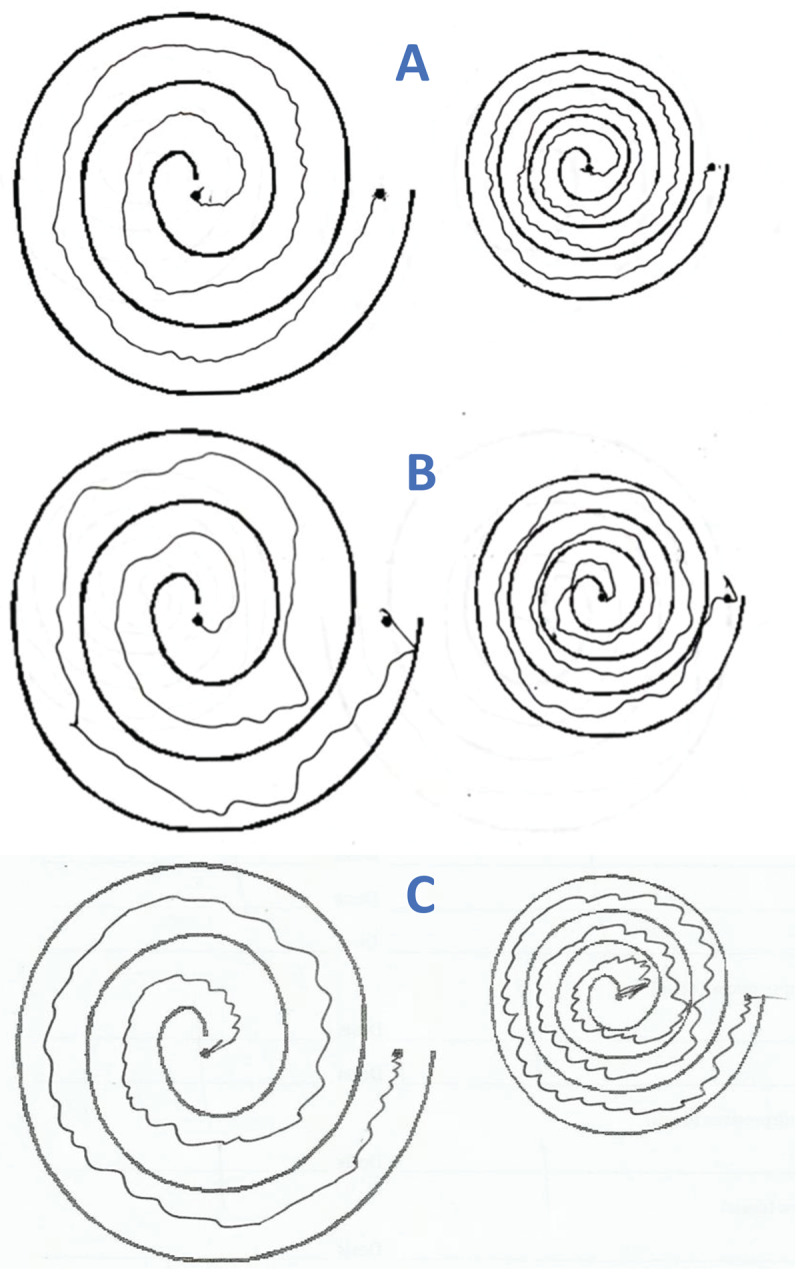
Examples of tremor in Archimedes spirals in two patients with Guillain-Barré, tremor has a jerky component without axes (**A** and **B**). Patient with essential tremor shows a regular tremor with axes **(C)**.

Sixteen patients (89%) were identified with upper limb tremor, one patient had tongue and voice tremor, this patient was receiving valproate for convulsive crises occurring during the acute phase of his GBS; however, tremor persisted with the same severity and distribution after a withdrawal for 72 hours. The patient did not have convulsive crises during this period and valproate was advised to be stopped. If a cut off value of 10 or more point in the total FTM-TRS was used, 6 out of 17 patients (35.3%) could be considered having a pathological tremor related to the GBS. Tremor distribution is shown in Table 2. No patient had facial, head, trunk and leg tremor. Tremor was mostly symmetric kinetic, of low amplitude, jerky, affecting the ability of drawing Archimedes spirals in the part B of the FTM-TRS (Figure 2, Table 3). Most patients with arm postural and kinetic tremor had oscillations at the wrist level; whereas one patient had finger tremor. Postural tremor had greater amplitude than kinetic tremor in 3, kinetic tremor had greater amplitude than postural tremor in 2, and it was equal in one patient out of 6 with hand tremor manifested with stretched arms and nose-finger task (Table 3). No difference was observed in FTM-TRS total score between patients with axonal GBS by neurophysiology (n = 7) and the rest of patients(n = 11), 7.17 ± 6.94 vs 13 ± 13.67 (*P* = 0.348). No statistically significant difference was observed between patients with the pure motor and sensory-motor variant 9.17 ± 7.58 vs. 15.20 ± 19.25 (*P* = 0.355).

**Table 2:** Frequency of tremor according to FTM-TRS Items.


FTM-TRS	N (%) OF PATIENTS WITH TREMOR/IMPAIRMENT

**Part A**	

Tongue tremor (R/P)	1/18 (5.5)

Voice tremor	1/18 (5.5)

Right upper limb tremor	

Rest	1/18 (5.5)

Postural	5/17 (29.4)

Kinetic	4/17 (23.5)

Left upper limb tremor	

Rest	1/18 (5.5)

Postural	4/16 (25)

Kinetic	6/16 (37.5)

**Part B**	

Writing tremor	7/17 (41.2)

Archimedes spirals drawing	

Right hand	11/16 (68.8)

Left hand	14/16 (87.5)

Straight line drawing	

Right hand	8/16 (50)

Left hand	12/16 (75)

Liquid pouring (R/L)	4/15 (26.6)

**Part C**	

Speaking	1/18 (5.5)

Feeding other than liquids	1/16 (6.25)

Bringing liquids to mouth	1/16 (6.25)

Hygiene	1/16 (6.25)

Dressing	2/16 (12.5)

Writing	7/16 (43.8)

Working	7/16 (43.8)


FTM-TRS: Fahn-Tolosa-Marin tremor rating scale. R/P: rest and postural. Binary data.

**Table 3:** Summary of tremor features on each patient who suffered Guillain-Barré syndrome.


CASE	SEX/AGE AT EVALUATION	TREMOR FEATURES	PART A	PART B	PART C	TOTAL	RESIDUAL WEAKNESS/SIGNS OF DENERVATION

1	M/41	Arch spirals 1+ (J)	0	2	0	2	None

2	M/37	Arch spirals 1+ (J)	0	2	0	2	None

3	F/20	UL postural 2+, kinetic 1+, writing 1+, Arch spirals 1+, finger tremor (J) P > K	8	7	2	17	None

4	M/57	Arch spirals 1–2+, (J)	0	5	0	5	Mild distal upper limb hypotrophy

5	M/28	None	0	0	0	0	None

6	F/38	Arch spirals 1+, straight line 1+	2	2	0	4	None

7	M/54	Arch spirals 1–2+, writing 1+, (J)	0	7	0	7	None

8	M/27	Tongue 1+, voice 3+, UL, rest 2+, postural 2+, kinetic 2+. Writing 4+, wrist tremor. P = K	17	4	27	48	Lower limb and N/E due to residual weakness, part B partially evaluated/distal upper limb muscle hypotrophy

9	M/26	Arch spirals 1–2+, straight line 1+, liquid pouring 1–2+, (J)	0	11	2	13	Mild residual upper limb weakness not interfering with tremor evaluation/distal upper limb muscle hypotrophy

10	M/49	Arch spirals 1+	–	2	–	2	Left upper limb and lower limbs N/E due to residual weakness/distal upper limb muscle hypotrophy

11	M/25	UL postural tremor 1+, Arch spirals 1+, straight line 1+, wrist tremor, P > K (J)	1	5	0	6	None

12	F/61	Arch spirals 1+, straight line 1+, writing 1+ (J)	0	5	1	6	None

13	F/49	UL kinetic 2+, Arch spirals 2+, straight line 1+, wrist tremor, K > P (J)	2	6	1	9	None

14	M/48	UL postural tremor 1+, Arch spirals 1–2+, straight line 1+, liquid pouring 1–2+, writing 2+, wrist tremor, K > P (J)	2	14	6	22	Mild distal upper limb muscle hypotrophy

15	M/54	UL postural 2+, kinetic 1+, Arch spirals 1–2+, straight line 1+, liquid pouring 1–2+, writing 2+, wrist tremor, P > K (J)	6	11	2	19	Mild residual upper and lower limb weakness not interfering with tremor evaluation

16	F/22	Arch spirals 1+	0	3	1	4	Distal upper limb muscle hypotrophy

17	M/50	Arch spirals 2–3+, straight line 2+, liquid pouring 1–2+, (J)	2	15	3	20	None

18	M/61	N/A	–	–	–	–	Severe residual upper and lower limb weakness, part B and C N/E


Arch: Archimedean; F: female; K: kinetic; N/A: not applicable; N/E: not evaluated; M: male; P: postural; UL: upper limbs. (J) jerky tremor. P > K: upper limb postural tremor had greater amplitude than kinetic tremor, K > P: upper limb kinetic tremor had greater amplitude than postural tremor, K = P: upper limb kinetic and postural tremor had similar severity.

In the multivariate linear regression model, including sex, age at onset, previous respiratory infection, previous intestinal infection, type of GBS (motor vs. motor-sensitive) cranial involvement, mechanical ventilation, cranial muscles involvement and worst Hughes score, as independent variables; only “days of hospitalization” showed a statistically significant positive association with the total FTM-TRS score (standardized Beta coefficient: 0.720, *P* = 0.001).

When comparing patients with GBS with 29 with ET, the former was younger (40.35 ± 13.5 vs. 58.8 ± 20.5,*P* = 0.002), more commonly male (62.5% vs. 17.24%, *P* = 0.001), and had lower scores in the part A (2.5 ± 4.5 vs. 12.1 ± 7.1, *P* < 0.001), part B (5.44 ± 3.6 vs. 9.48 ± 6.6,*P* = 0.011), part C (2.8 ± 6.6 vs. 7.1 ± 5.7, *P* = 0.029) and total FTM TRS: 10.94 ± 11.84 vs. 28.62 ± 17.47, *P* = 0.001. No patient with GBS related tremor showed a clear axis in the Archimedes spirals; whereas this feature was observed in 31% of patients with ET (*P* = 0.008) (Figure 2).

## Discussion

In this study, we assessed the frequency and clinical features of tremor in patients following an episode of GBS. We observed a high frequency of postural and kinetic upper limb tremor. This tremor caused a functional impact, which added disability to the muscle weakness still suffered by a third of our patients.

This tremor is reported to have some features of ET, predominantly kinetic, involving the upper extremities and does not seem to modify with weight loading [16]. One study enrolling 5 patients recovering from GBS, reported that patients had low amplitude, fast (8–10 Hz) tremor with a slight jerky component [17]. In that study tremor was reported to appear 3 months to 5 years after onset of GBS, basically when patients had regained normal muscle strength and elicitable deep tendon reflexes [17]. However, we were not able to identify the time onset of tremor in our patients and a proportion of them still have muscle weakness at tremor evaluation.

We did not assess for tremor before the onset of GBS, as all patients first went to the hospital owing to the GBS. In a population-based study on tremor drawing Archimedes spirals in 1158 normal individuals, between 40 and 98 years of age, in the Faroe Islands; the authors reported tremor in 98.9% of cases [18]. In general, tremor was mild in this study, but moderate tremor was observed on the left hand in males over 70 years [18]. In our study, using a threshold of 10 or more point in the total FTM-TRS, 35.3% of patients could be considered as having a pathological tremor related to the GBS.

Tremor is common in inflammatory neuropathies such as paraproteinemic IgM neuropathy, CIDP, and multifocal motor neuropathy with conduction block. In those cases, tremor was mainly kinetic and affecting the upper extremities [4]. Antibodies directed against anti-neurofascin-155 have been linked with CIDP and tremor in 42% of cases [19]: this tremor is usually severe, postural and mainly affects the upper limbs, but can be observed in the tongue and is usually associated with ataxia, CNS demyelination and poor response to IVIg [19,20,21]. Other antibodies, including anti-neurofascin-140/186, anti-contactin-1, anti- contactin-1 associated protein (CASPR-1) have also been associated with CIDP and tremor in selected patients [22]. These patients differ from ours, mainly in tremor severity or amplitude; while patients with CIDP usually present with moderate to severe tremor; patients from this cohort showed mild tremor that became evident with drawings.

The mechanism of tremor in patients with peripheral neuropathies is debated. A study in patients with CMT and tremor, showed no evidence of cerebellar dysfunction, suggesting that peripheral mechanisms were implicated in tremor generation in such cases [23]. While other studies have shown impaired responses to classical eyeblink conditioning and paired associative stimulation in patients with inflammatory neuropathy and tremor compared with those without tremor [24]. This data supports a role of central mechanisms, involving the cerebellum, in the pathogenesis of tremor. As neuropathic tremor may have various clinical presentations resembling ET, enhanced physiological-like tremor or cerebellar tremor, the pathophysiology may be heterogenous with diverse central and peripheral mechanisms contributing on each case [25]. Moreover, the role of an axonal vs demyelinating nerve disorder in the pathogenesis of this type of tremor is unclear. In a study of 89 patients with polyneuropathy and documented etiology, tremor was observed in 74% of cases by objective evaluation, no relationship was observed with type of neuropathy (axonal or demyelinating) [26]. Suggesting that both types of nerve damage may be implicated in tremor pathogenesis.

It is unclear which variables related to the severity and demographics of GBS may predict tremor, as there is no clear cut off value to consider a pathological tremor, we assessed independent variables and considered the total FTM-TRS as the dependent variable. Only “days of hospitalization” showed a positive significant association (*P* = 0.001). It is possible that patients with longer hospitalization time have a longer GBS damage, but they are exposed to potential more insults such as medications, ischemia, infections, etc. that may eventually contribute to tremor [27].

Our study has limitations, first of all, the number of studied patients is reduced and we were not able to stablish correlations or risk ratios between tremor and clinical features of GBS. We did not measure anti-ganglioside antibodies. Another limitation is the lack of neurophysiology studies to assess tremor frequency and loading effect and fully characterize the type of GBS. Although no statistical difference was observed in FTM-TRS total score between patients with axonal type GBS and those not tested with neurophysiology and pure motor vs sensory motor GBS, the number of studied patients is limited to draw definite conclusions related to the role of the GBS type and risk of tremor. Moreover, we did not test a particular pharmacological measure for the treatment of GBS tremor. Other studies have reported benefit for neuropathic tremor with pregabalin, but medications from other pharmacological groups may be helpful [28].

## Conclusions

Tremor was observed in 89% of patients following GBS in our cohort. Tremor was predominantly kinetic, with a jerky character, affecting mainly the upper extremities. Although relatively mild compared with patients with ET, it represented an additional feature contributing to the functional impact in patients with GBS.

## Data Accessibility Statement

Data is available on request to the authors.

## Abbreviations

AIDP: acute inflammatory demyelinating polyradiculoneuropathy

AMAN: acute motor axonal neuropathy

AMSAN: acute motor sensory axonal neuropathy

CASPR-1: contactin-1 associated protein

CMT: Charcot-Marie-Tooth

CIDP: chronic inflammatory demyelinating polyradiculoneuropathy

ET: essential tremor

FTM-TRS: Fahn-Tolosa-Marin tremor rating scale

GBS: Guillain-Barré syndrome

IVIg: intravenous immunoglobulin

mRS: modified Rankin Scale

MRC: Medical Research Council

PE: plasma exchange

SNRIs: serotonin and norepinephrine reuptake inhibitors

SSRIs: selective serotonin reuptake inhibitors
